# The effect of column purification on cDNA indirect labelling for microarrays

**DOI:** 10.1186/1746-4811-3-9

**Published:** 2007-06-27

**Authors:** M Lia Molas, John Z Kiss

**Affiliations:** 1 Department of Botany, Miami University, Oxford, OH 45056, USA

## Abstract

**Background:**

The success of the microarray reproducibility is dependent upon the performance of standardized procedures. Since the introduction of microarray technology for the analysis of global gene expression, reproducibility of results among different laboratories has been a major problem. Two of the main contributors to this variability are the use of different microarray platforms and different laboratory practices. In this paper, we address the latter question in terms of how variation in one of the steps of a labelling procedure affects the cDNA product prior to microarray hybridization.

**Results:**

We used a standard procedure to label cDNA for microarray hybridization and employed different types of column chromatography for cDNA purification. After purifying labelled cDNA, we used the Agilent 2100 Bioanalyzer and agarose gel electrophoresis to assess the quality of the labelled cDNA before its hybridization onto a microarray platform. There were major differences in the cDNA profile (i.e. cDNA fragment lengths and abundance) as a result of using four different columns for purification. In addition, different columns have different efficiencies to remove rRNA contamination. This study indicates that the appropriate column to use in this type of protocol has to be experimentally determined. Finally, we present new evidence establishing the importance of testing the method of purification used during an indirect labelling procedure. Our results confirm the importance of assessing the quality of the sample in the labelling procedure prior to hybridization onto a microarray platform.

**Conclusion:**

Standardization of column purification systems to be used in labelling procedures will improve the reproducibility of microarray results among different laboratories. In addition, implementation of a quality control check point of the labelled samples prior to microarray hybridization will prevent hybridizing a poor quality sample to expensive micorarrays.

## Background

Microarray technology has become widely used to evaluate global gene expression. Despite the increasing reliance on this technology, the poor reproducibility of microarray data among laboratories and across platforms is still a major concern [1]. Currently, there are a variety of platforms available, ranging from cDNA to oligonucleotide microarrays, to customized arrays, and to commercially developed microarrays. In addition, there is a large diversity in the procedures used by different laboratories for RNA manipulation and labelling as well as software used to analyze the output data. As a result of these multifaceted approaches to assess gene expression, comparisons of the final results are complex and many times not reproducible [1].

A number of recent studies have addressed the complexity of microarray variability [2-8]. The use of different microarray platforms and different practices in laboratories (i.e., lab-to-lab variability or "lab effect") emerges as a major source of variability. In this regard, preparation of samples and labelling procedures increase the technical variation and significantly affect the quality and reproducibility of the data [9,6]. For example, procedures such as RNA extraction have proven to significantly increase the technical variability in microarray data [10]. Hence, standardizing methodologies and establishing quality control check points to ensure reproducibility are recognized as critical in microarray experiments. In an attempt to reach consensus on the generation, analysis and application of microaray data, the **m**icro**a**rray **q**uality **c**ontrol (MAQC) project evaluated the reproducibility in microarray results among platforms [7,8]. Comparison of quantitative gene expression of two commercial human RNA samples was considerably consistent among platforms compared to reports in previous studies. These studies conclude that using common tools for data analysis, applying quality control criteria, and standardizing the data report provide confidence in the consistency of gene expression data.

Microarray technology utilizes various procedures to label mRNA samples [11]. Indirect labelling protocols use amino-substituted nucleotides incorporated during a reverse transcription reaction into cDNA. A nucleotide analog carrying a chemically reactive amine group (i.e. amino-allyl substituted dUTP) is later conjugated to a fluorescent dye [12]. The resulting primary amine group from the amino-allyl dUTP (aa dUTP) is conjugated to a succinimidyl ester cyanine (Cy) fluorescent molecule at the 5- or 3- (Cy3 and Cy5) carbon of the pyrimidine base. For the cDNA and oligonucleotide microarrays, Cy3 and Cy5 are commonly used fluorescent dyes that are exited by different wavelengths of light. As a result, they can be used in combination, one labelling a control or reference sample and the other labelling the treatment or test sample. Gene expression can be measured by calculating the ratio of the two different cyanine dye fluorescence intensities detected after combining the two labeled cDNA samples and hybridizing to a microarray platform.

The quality of the initial RNA sample is the first step to ensure successful cDNA and cRNA synthesis before microarray hybridization. One of the most effective tools for characterizing RNA integrity is capillary electrophoresis, in which RNA degradation is indicated by an altered 28S/18S ribosomal RNA (rRNA) signal ratio. Auer and colleges established a "degradation factor" utilizing data obtained from a RNA assay of the Agilent Bioanalyzer as a more quantitative approach [13]. This approach calculates a ratio between the 18S ribosomal peak area and the average of the peaks smaller than 18S that are indicative of degradation. The authors show that if we compared two samples, intact and degraded RNA, respectively, up to three quarters of differential gene expression measured was due solely to differences in RNA integrity between two samples [13].

The percentage of intact rRNA present in the original sample has been suggested as an important criterion of a non-degraded RNA sample [14]. In the same way, the median size of the cDNA and cRNA synthesis products is a good indicator of the quality of the sample. cDNA and cRNA synthesis products with median sizes of 2.0 and 3.0 kb, respectively, were found suitable for microarray hybridization [14].

This study shows one source of technical variability during sample preparation prior to microarray hybridization by analyzing a cDNA labelling procedure. Specifically, the aminoallyl indirect labelling protocol was evaluated by using four different ultrafiltration devices for purifying fluorescently labeled cDNA products. We found that different systems of column purification used during the labelling procedure resulted in different cDNA populations. In addition, contamination with rRNA is present in some cases. Consequently, assessing the quality of cDNA samples immediately prior to microarray hybridization is an important checkpoint that will help diminish technical variability.

## Results

**Figure 1:**
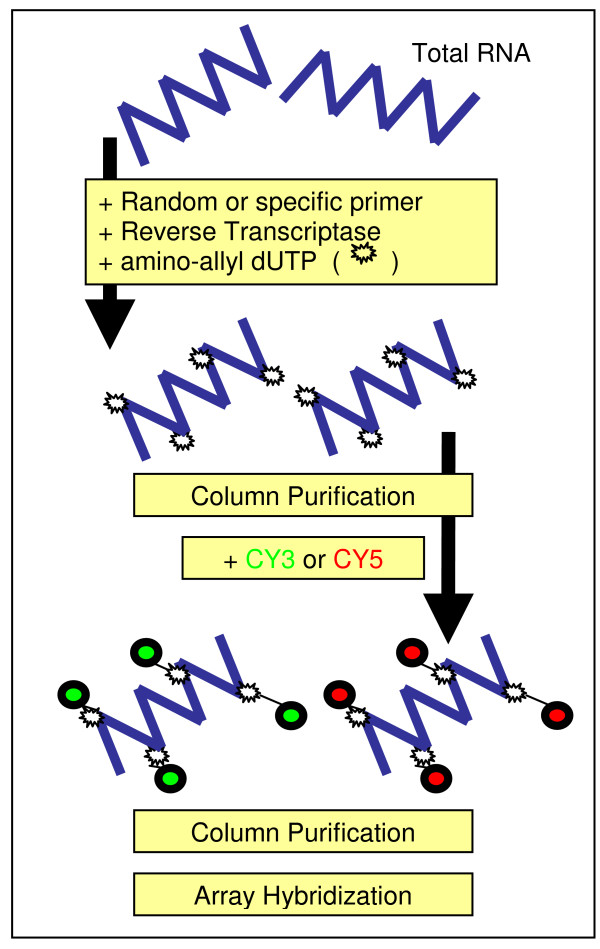
**Principle of cDNA indirect labelling method, starting form total RNA sample**. Total RNA is labeled by reaction of Cy-dyes with amino-allyl dUTP incorporated during the first cDNA strand synthesis (cDNA indirect labeling). The same columns for cDNA purification were used before and after labeling.

In the present study we analyzed the amino-allyl indirect labelling method to generate cDNA probes labeled with Cy5 (Figure 1). Using a standard procedure, we contrasted the performance of four different chromatographic columns for cDNA purification. In brief, we used AutoSeq G-5-columns (Amersham), Qiaquick PCR purification kit (Qiagen), Microcon YM-30 centrifugal filter (Millipore), and DNA Clean and Concentrator-5 (Zymo Research). We designate each column as column A, B, C and D, respectively. After labelling the sample, we analyzed the quality of the Cy5-labeled cDNA product by two electrophoretic methods, the Agilent 2100 Bioanalyzer and agarose gel electrophoresis.

**Figure 2:**
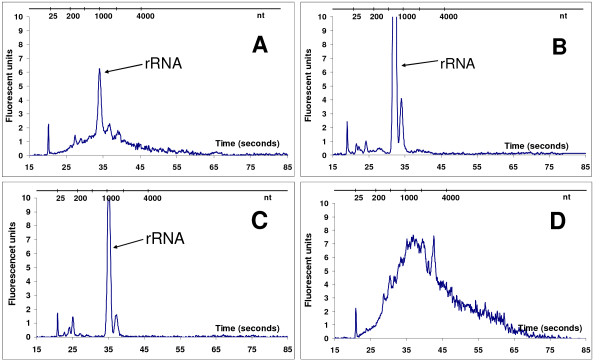
**Chromatograms of micro-capillary electrophoresis from Cy5-cDNA samples**. Chromatograms show different profile after using different purification methods (as indicated in the method section) in an indirect labelling protocol. rRNA contamination is marked by arrows. Above each graph nucleotide size is indicated (nt). Profiles of one representative experiment are shown. Experiments were replicated a minimum of three times.

Cy5-cDNA samples purified with the four different purification columns have cDNA electropherogram patterns that are significantly different (Figure 2). Analysis of the cDNA patterns shows that different cDNA fragment lengths and abundance are obtained based on the method used for Cy5 cDNA purification (Figure 2). In addition, the rRNA contamination in the cDNA sample is readily identified with the Agilent Bioanalyzer (indicated in Figure 2).

Results with column A show a smooth Cy5-cDNA profile, concentrated in the middle region and few individual peaks. Columns B and C present similar profiles in the electropherograms, both of these have more defined peaks with flat baselines and relatively flat valleys between the peaks (Figure 2B and 2C). Both columns B and C have significant contamination with rRNA. On the other hand, column D presents an evenly distributed profile, and the absence of individual peaks. Column D performed very well in terms of having little rRNA contamination and high cDNA yield.

To better characterize the performance of each purification device, the median size of the cDNA population, the presence of rRNA in the sample, and the cDNA yield per column was measured (Table 1). The three parameters evaluated showed significant variability. Overall, columns with high yield present high rRNA content. cDNA median size differs among columns indicating that, even when they have a wide range for product size isolation, they act more efficiently in separating certain sizes of cDNA transcripts.

**Table 1:** cDNA yield (ng), presence of rRNA (%) and cDNa median size (nt) of labeled cDNA using four different columns for cDNA purification

	**Yield (total ng)**	**rRNA (%)**	**cDNA median size (nt)**
Column A	399 ± 19.9	42.2 ± 11.5	2075 ± 37.4
Column B	696 ± 93	61 ± 1.04	1483 ± 132.1
Column C	292.8 ± 43	60.6 ± 1.71	1241 ± 394.9
Column D	184 ± 9.2	2.9 ± 1.19	3017 ± 74.5

**Figure 3:**
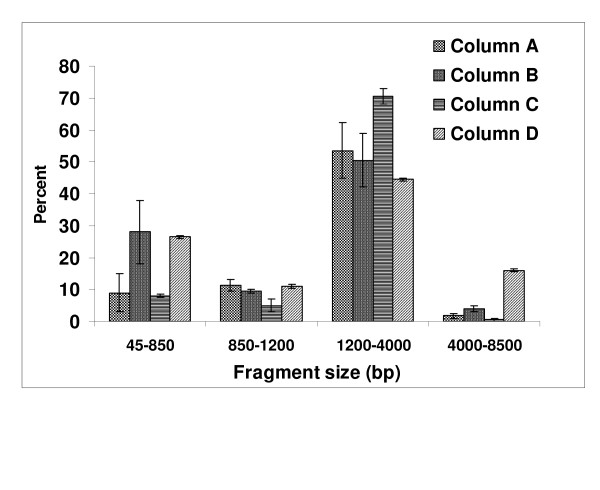
**Comparison of Cy5-cDNA profile by regions using four chromatographic methods for Cy5-cDNA purification**. Each region is quantified as area under the curve and expressed as a percentage. rRNA is not included in this analysis. Each value represents the mean and SE of three biological replicates.

To examine the variability in the cDNA patterns, we divided the electropherograms into regions based on cDNA size (in bp). These regions were used to quantify the percentage of cDNA fragments (i.e., transcripts) present in each region. The amount of transcript present in each region was calculated by integrating the area under the curve in each region in relation to the total area. rRNA fragments were not included in this analysis since they have been quantified in Table 1. Figure 3 shows a comparison per region of the cDNA fragment length obtained from the columns evaluated. High variability in each region can be observed based on the type of column used.

**Figure 4:**
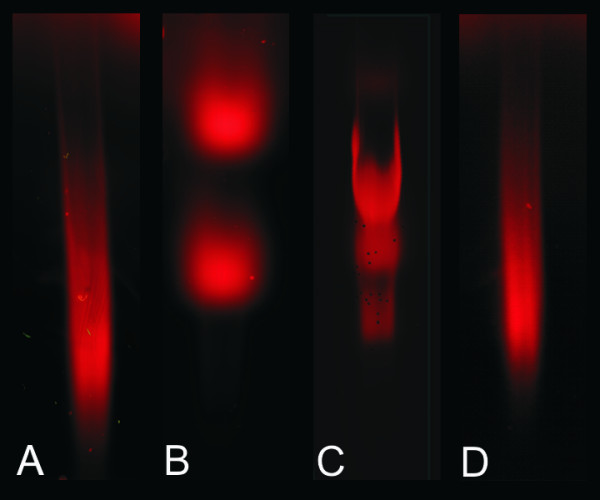
**Conventional analysis of Cy5-cDNA separated based on size using a 1 % (w/w) TEA agarose gel**. Red fluorescence was measured using Axon GenePix 4000 scanner with Gene Pix Pro 6.0 software

Labeled Cy5-cDNA analyzed by agarose gel electrophoresis also showed different Cy5-cDNA populations visualized as different smear profiles on the gel (Figure 4). Evaluation of the Cy5 cDNA samples prior to microarray hybridization via gel electrophoresis does not provide information about rRNA contamination since its presence, if any, is overlapped by Cy5-cDNA fragments migrating in the same region. Hence, discrimination between rRNA and cDNA is not possible using agarose gel electrophoresis.

**Figure 5:**
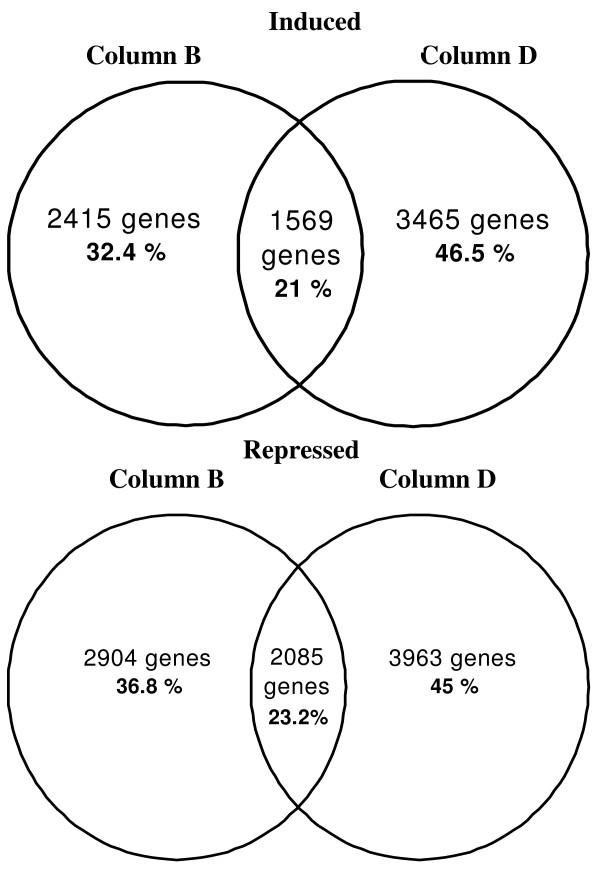
**Number of genes defined as responding to 1 h of red light in 7 day old Arabidopsis seedlings using two column purification devices**. Venn diagrams show the number of genes up- and down-regulated > 2-fold by red light of as detected by column B (left) and column D (right). Number of genes detected per column and overlapping between columns is expressed as percentage (%) of the combined gene list of up- and down-regulated respectively. Results form one independent slide is shown.

Microarray analyses were performed employing labeled cDNA with the standard procedure using two columns of contrasting cDNA pattern (i.e. column B and D). The genes regulated at least 2-fold (log2 ratio of red/dark signal > 1) showed similar percentage of the total number of genes in both experiments (13.9 % and 17.3 % as up-regulated for column B and D respectively and 17.2 % and 19.1 % down-regulated in column B and D respectively). However, the number of genes detected for both column B and D overlap only 21 and 23 % for up- and down-regulated genes respectively (Figure 5). These results indicate that both purification devices isolate cDNA suitable for hybridization but that this is not enough to reproduce the same microarray results.

## Discussion

### Different purification systems generate different Cy5 cDNA population

Cy5-cDNA samples purified with the four different methods show cDNA electropherogram patterns that are significantly different (Figure 2 and 3). Analysis of the cDNA pattern shows that different cDNA fragment lengths and abundance are obtained based on the method used for Cy5 cDNA purification (Figure 2 and 3). In addition, the yield per column and the efficiency to remove rRNA from the cDNA sample is noticeably different among the columns (Table 1).

Different methods for Cy-cDNA purification generate different cDNA populations and this discrepancy might be related to variables other than the purification method employed. For example, columns C and D both use silica-based matrices to isolate Cy-cDNA. In this case, cDNA binds to a matrix of silica in the presence of a high concentration of a chaotropic salt while other molecules are not retained. Even though columns C and D employ the same mechanism for cDNA isolation, they differ in the Cy-cDNA pattern obtained after purification, the cDNA median size, and on their effectiveness to eliminate rRNA (Table 1 and Figure 2).

On the other hand, columns A and B use particle size as the principle for isolation. Column A separates Cy-cDNA by a gel pore exclusion which retains the unincorporated Cy dye in the gel matrix and allows the larger Cy-cDNA fragments to flow through the column. In column B, however, the cDNA molecules are retained on a cellulose membrane with pore size as the separating mechanism; the unincorporated dye-labeled nucleotides flow through the membrane while the larger Cy-labeled cDNA is retained. These two columns give different Cy-cDNA profiles, indicating that the method employed for separation, even when they use the same principle, results in Cy-cDNA populations of different composition (i.e. fragment length and abundance) as observed in the electropherogram and gel images.

In light of these results, column performance does not seem to depend entirely on the principle of separation they use. Moreover, we can find some similarities between columns A and D in the cDNA profile, even though both columns are based on different principles of separation. Hence, aspects other than the methods of purification itself are affecting the final results in each case. Variables related to the optimization of the column should be considered in order to identify the best performing column in each particular procedure.

Microarray analysis performed with columns B and D showed that both devices were able to produce adequate cDNA sample to perform hybridization and an acceptable number of genes [15,16] were detected as up- and down- regulated more than 2-fold. However, a comparison of the genes detected as regulated more than 2-fold in both of the columns tested, overlap on average of 22 % (Figure 5). These results clearly show that the column used for cDNA purification in the labelling process ultimately affect the results in microarray experiments.

### Detection of rRNA in cDNA sample

Three of four column methods evaluated in this study showed considerable amount of rRNA (Figure 2, Table 1). rRNA will contribute to absorbance readings at 260 nm (A260) used to calculate mRNA and/or cDNA quantity. Consequently, when using A260 values to calculate mRNA and/or cDNA quantity, the presence of rRNA in the sample may provide inaccurate results.

In this study, we used oligo dT primers to synthesize cDNA from total RNA during reverse transcription, and for this reason we did not expect to have cDNA made from rRNA. However, many protocols for both indirect and direct labelling use random primers. When random primers are used, at least some rRNA may be transcribed, producing Cy-labeled cDNA, and this cDNA from rRNA may hybridize to complementary sequences in the array. If this were the case, erroneous differential expression data would potentially be obtained, leading to incorrect interpretation of the microarray results.

rRNA and low molecular weight RNA frequently contaminates mRNA preparations [11]. During the process of purification, small rRNA is non-specifically bound to the column matrix and, additionally, some rRNA's are bound to and co-purify with the mRNA [17]. Considering that rRNA constitutes more than 80% of the total RNA, it is difficult to obtain its complete degradation. Alkaline hydrolysis, the method used in this protocol to degrade rRNA, incorporates a molecule of water into the phosphodiester bonds constituting both RNA and DNA primary structure. These ester bonds are hydrolyzed; this process rapidly destroys RNA and more slowly destroys DNA. In the particular case of this study, a "compromise situation" is taking place in that rRNA has to be broken down to small fragments, but the cDNA present in the sample has to be preserved from hydrolysis. This delicate balance is controlled by the time of exposure to high pH at elevated temperature. It is plausible then, that we may have an incomplete digestion of the rRNA. This would render large fragments of rRNA instead of small size fragments (ideally a few nucleotides length) thus making it more difficult to separate from cDNA fragments.

While information about rRNA contamination can be obtained from the Agilent Bioanalyzer electropherograms, no data addressing rRNA contamination can be obtained by gel electrophoresis. Since rRNA and cDNA overlap in length and migrate in the same region of the gel, rRNA would be masked by the Cy-cDNA labeled fragments. This fact makes agarose gels of limited use in terms of detection of such contamination.

## Conclusion

This study shows that different methods for Cy-cDNA purification generate different cDNA populations for hybridization of microarray platforms. Discrepancies in column performance may be related to variables other than the purification method itself. Results from microarray experiments confirm that the use of different columns for purification in an indirect labelling protocol may produce different gene expression data. Hence, the purification method of choice used in cDNA labelling procedures prior to microarray hybridization is an important parameter to consider.

## Methods

### RNA preparation

Total RNA was extracted from leaves of light grown 10 day old plants of *Arabidopsis thaliana *(ecotype Landsberg erecta) using the RNeasy^® ^Mini Kit (Qiagen, Valencia, CA, USA). Residual DNA was removed by performing an on-column digestion using a DNA-free TM kit (Ambion, Austin, TX, USA) according to manufacturer's instructions. RNA concentration and purity were estimated with the NanoDrop ND-1000 spectrophotometer (NanoDrop, Wilmington, DE, USA) and quality further assessed with the Agilent 2100 Bioanalyzer (Agilent, Santa Clara, CA, USA). One single total RNA extraction per biological replicate was performed and subsequent used for labelling reaction. A single pool of total RNA was reverse transcribed and divided into four aliquots for purification and labelling. Three independent biological replicates were performed using leaves from 10-day-old plants.

### Indirect Cy5 incorporation

Total RNA from one single extraction was reverse transcribed and separated in four equal aliquots. Each aliquot (containing 10 μg of total RNA) was labelled according to the TIGR protocol [18] with modifications (schematic protocol in Figure 1). Briefly, we used SuperScript II reverse transcriptase (Invitrogen, Carlsbad, CA, USA) and oligo dT primer to synthesize the first-strand cDNA. A chemically reactive nucleotide analog (amino allyl-dUTP) was incorporated into cDNA. After reverse transcription, the mRNA template was hydrolyzed by alkaline hydrolysis and the cDNA purified to remove free nucleotides and oligomers. In this first purification step we employed four different chromatographic methods: A) CyScribe GFX™ PCR DNA (Amersham, Piscataway, NJ, USA), B) Qiaquick PCR purification kit (Qiagen, Valencia, CA, USA), C) Microcon YM-30 centrifugal filter (Millipore, Billerica, MA), and D) DNA Clean & Concentrator-5 (Zymo Research, Ontario, Canada). The purified cDNA was then post labeled with the reactive form of Cy5-NHS ester, which bonded to the modified nucleotide amino allyl-dUTP. The reaction was quenched with 4.5 μl of 4 M hydroxylamine for 15 min at room temperature. A final step of purification was then performed resulting in purified CyDye-labeled cDNA, which was ready for hybridization. The same columns for purification were used in the first and second purification steps (see Figure 1). Purifications were performed in parallel for the four columns. cDNA from the first purification was dried and stored at -20 C to the next day, when the second purification was performed.

### Cy5 detection by the Agilent 2100 Bioanalyzer

One μl of each Cy5 labeled cDNA sample was loaded onto an Agilent RNA 6000 Nano LabChip (RNA LabChip, Agilent Technology, Santa Clara, CA) and assessed with mRNA Nano Assay. cDNA fragment size, rRNA contamination, and overall amount of fluorescence were evaluated. The detected fluorescence is the addition of the intercalating dye (provided in the Agilent RNA Nano Assay) and Cyanine 5 incorporated into the labeled cDNA. The Smear Analysis tool of the Agilent Bioanalyzer software was used to quantify the percentage of cDNA transcripts present in each region.

For the rRNA samples, the Cy3 was prepared for the hybridizations. We observed a lack of significant contribution to the overall fluorescence on the Bioanalyzer by Cy3-labeled samples (data not shown). Cy3 does not fluoresce in the range of the Bioanalyzer laser, and any contribution to the outcome of the test would be minimal and consistent across all samples.

### Gel electrophoresis

Five μl of each Cy5 labeled cDNA and 5 μl 30 % (v/v) glycerol was loaded onto a 1% (w/v) TAE agarose gel. Loading dye was run in an independent lane to prevent interference when Cy5 labeled cDNA is measured. The electrophoretic runs were performed at 90 V for 45 min. Red fluorescence (650 nm) of Cy5 was detected using the Axon GenePix 4000b scanner with Gene Pix Pro 6.0 software (Molecular Devices, Sunnyvale, CA, USA)

### Microarray analyses

60 μg of total RNA from 7 day old dark-grown seedlings exposed to one our red light (i.e. control dark vs. 1 h. red light experimental) were reverse transcribed and labeled as indicated in previous section. Column B and D were used for cDNA purification in two separate experiments conducted in parallel (i.e. total RNA extraction, cDNA labelling, and hybridization of samples were performed at the same time using the same conditions). In the figure 5, microarray hybridization of one biological replicate was performed.

Fluorescently labeled cDNAs were mixed with hybridization solution (SliderHyb Survey, Ambion) and hybridized to the microarray slide overnight in a 55°C water bath. Samples were hybridized in separate long oligonucleotide microarray slides (University of Arizona) containing 29,000 oligonucleotide array elements. A complete listing of the genes on this chip is available on reference 19. cDNA microarray slides were prepared according to the manufacturer's instructions [20].

The cDNA microarray slides were scanned with a GenePix 4000b scanner using laser excitation at 635 and 532 nm at 100% PMT sensitivity. Spot intensities were quantified using Axon GenePix Pro 6.0 image analysis software. The net intensities for each channel and channel ratios were measured using this software with the ratio method (median intensity). Then, normalization based on median of intensities was performed in the two experiments.

## Abbreviations

amino-allyl dUTP (aa-dUTP)

cyanine 5 or 3 (Cy5 and Cy3)

complementary DNA (cDNA)

ribosomal RNA (rRNA).

## Competing interests

The author(s) declare that they have no competing interests.

## Authors' contributions

MLM performed the experiments and drafted the manuscript. JZK supervised the study and participated in writing the manuscript. Both authors read and approved the final manuscript.
